# Kinetics of Parasite-Specific Antibody and B-Cell-Associated Gene Expression in Brown Trout, *Salmo trutta* during Proliferative Kidney Disease

**DOI:** 10.3390/biology10121244

**Published:** 2021-11-28

**Authors:** Saloni Shivam, Mansour El-Matbouli, Gokhlesh Kumar

**Affiliations:** 1 Clinical Division of Fish Medicine, University of Veterinary Medicine Vienna, 1210 Vienna, Austria; salonis@staff.vetmeduni.ac.at (S.S.); mansour.el-matbouli@vetmeduni.ac.at (M.E.-M.); 2 Central Marine Fisheries Research Institute, Karwar 581301, India

**Keywords:** salmonids, parasite sacs, malacosporean, ELISA, anti-*T. bryosalmonae* antibody, B-cell-mediated response

## Abstract

**Simple Summary:**

The parasite *Tetracapsuloides bryosalmonae* causes proliferative kidney disease in salmonids. In general, antibodies and B cells play important roles in host defense during chronic infections. In this work, we studied the antibody and B-cell-associated gene expression of brown trout during the course of *T*. *bryosalmonae* infection. Our results show that antibody responses mounted by brown trout change over time and are maintained at a low level throughout the infection duration. This might be reflective of the host strategy to limit parasite numbers for its survival. Additionally, the expression of genes having important roles in the development, differentiation and signaling of B cells was analysed in the kidney and spleen of infected brown trout from preclinical to post-clinical phases. Our findings indicate that the expression of B-cell-associated genes modulate during the course of parasite development which is suggestive of their critical role for protecting the host against this parasitic invasion. This study brings in important knowledge about the antiparasite antibody and B-cell-associated gene response in infected brown trout, which could be instrumental in developing therapeutic and prophylactic measures against this parasite in future.

**Abstract:**

*Tetracapsuloides bryosalmonae*, a myxozoan endoparasite often causes chronic infection in brown trout. Antiparasite immunity mediated by antibodies and B cells is known as an important determinant of host survival and parasite proliferation during chronic infections. Accordingly, studying their time course during proliferative kidney disease (PKD) might be helpful in improving our understanding of its chronic nature. Therefore, we conducted this study to examine parasite specific serum antibody and B-cell-mediated response in laboratory-infected brown trout at different time points. Brown trout were exposed to the spores of *T. bryosalmonae*, derived from infected bryozoans. Samples were collected at different time points and processed for indirect ELISA, histopathology, and qRT-PCR. *T. bryosalmonae* specific antibody was detected at 4 weeks post exposure (wpe) and it persisted until 17 wpe. Additionally, the expressions of C4A, CD34, CD79A, BLNK, CD74, BCL7, and CD22 were differentially regulated in the important immune organs, kidney and spleen. To our knowledge, this is the first study addressing anti-*T. bryosalmonae* antibody response in brown trout at different time points. The results from this study provide valuable insights into the processes leading to changes in B cell development, inflammation and antibody production during the course of PKD in brown trout.

## 1. Introduction

Brown trout (*Salmo trutta*) are a dominant wild fish species in Europe, and are globally introduced into suitable environments. Of late, an increasing number of reports suggest that wild brown trout populations are declining in European nations such as Austria and Switzerland. Proliferative kidney disease is considered one of the main drivers for their decline, due to its high prevalence in wild brown trout populations [1,2,3,4,5]. The association of this disease with fish stock falloffs stems from the fact that incidences and severity of PKD elevate with elevated water temperatures, resulting in higher mortality. Additionally, infected brown trout transmit the spores of *T. bryosalmonae* to bryozoans [6], and both hosts act as reservoirs for the parasite dispersal in the wild [7].

PKD is widespread among salmonids in Europe and North America [1]. The disease is caused by the myxozoan endoparasite *T. bryosalmonae* and affects susceptible fish of all ages [8]. The major clinical manifestations of this disease in infected fish include renal hyperplasia (kidney swelling) and splenomegaly (spleen enlargement) resulting from intense inflammatory response in these tissues. During advanced pathogenesis, pale gills, indicative of anaemia, are frequently observed [9].

*T. bryosalmonae* undergo a complex life cycle with the bryozoans as primary and the salmonid fish as secondary host. The spores released from infected bryozoans infect susceptible fish through gills [10]. Subsequently, the parasites migrate to the primary target organ, the kidney, as well as other organs mainly spleen and liver through blood [11]. Interestingly, this parasite can infect and develop clinical symptoms in many salmonids but can complete its life cycle only in some of them, such as brown trout (*Salmo trutta*) and brook trout (*Salvelinus fontinalis*) [12]. In rainbow trout, the parasite infection results in severe clinical symptoms. However, infected rainbow trout do not shed viable parasite spores infective for bryozoans, thus halting the parasite life cycle and, consequently, rainbow trout are referred as dead-end hosts [12]. Parasites are transmitted to fish through spores released from infected bryozoans, and in turn, bryozoans are infected by spores released from infected fish, but fish-to-fish transmission of the parasite does not occur [13].

B cells and antibodies are central to immunopathogenesis in hosts during parasitic infections. In addition to producing antibodies, B cells are required for antigen presentation and secretion of cytokines and chemokines, which mediate many pathways in the immune response [14,15]. B cells are characterised by the presence of B-cell receptors (BCR) which are required for B-cell survival and development, as well as antibody production [16]. The important roles of antibodies are well demonstrated during mammalian parasitic infections such as *Plasmodium* [17], *Trypanosoma cruzi* [18], *T. brucei gambiense* [19] and *Necator americanus* [20]. B cells are essential for the development of Th2 cell response for protection as demonstrated against *Leishmania* [21] and helminths infections [22]. Besides, many studies reported the importance of B cells and antibodies in reducing parasite burden [23,24,25,26].

While in fish hosts, protective immune responses following infections are reported against several parasites [27], unlike mammals, information on precise functions of antibodies and B cells is rather restricted. Involvement of antibodies and B cells has been demonstrated in some infections such as *Enteromyxum leei* [28], *Enteromyxum scophthalmi* [29] and *Ceratomyxa shasta* [30]. During advanced stages of *T. bryosalmonae*-induced PKD pathogenesis, B-cell activity decreases in the dead-end host rainbow trout [31,32,33]. In brown trout, this parasite develops chronic infections [34], and the infected fish are capable of releasing viable spores for several years [7]. There are some reports of gene expression studies in the kidneys of brown trout in response to *T. bryosalmonae* [33,35]. Recently, a global transcriptome analysis of the posterior kidney of brown trout during PKD was performed to visualise a broader picture during host–parasite interaction [36]. Despite these significant advances, there is no report of systematic serum antibody response of brown trout against *T. bryosalmonae*. Besides, dominant B-cell response is reported during PKD in brown trout [33], yet gaps remain in its functional understanding. In the previous study, we identified some B-cell-associated genes with roles in haematopoiesis and B-cell receptor signalling to express variably during PKD pathogenesis [36]. This study was thus designed to quantify antiparasite antibody levels in the blood of *T. bryosalmonae*-exposed brown trout using indirect ELISA and to investigate systematic B-cell-associated gene expression in kidney and spleen at different time points.

## 2. Materials and Methods

### 2.1. Fish Sampling

The details of the experimental exposure of brown trout to the *T. bryosalmonae* have been described in our previous study [36]. Briefly, brown trout (*n* = 69, 12 ± 2 cm) were exposed to the spores of *T. bryosalmonae* released from the laboratory-infected bryozoans [6]. Fish were maintained in 100 litre capacity aquaria with continuous water flow through system at 15 ± 1 °C with sufficient feeding. Blood, posterior kidney and spleen were collected from exposed and unexposed control fish, individually at 2, 4, 6, 8, 10, 12, and 17 weeks post exposure (wpe). Approximately 0.6–0.8 mL blood was drawn from the caudal vein of individual fish, and allowed to clot at room temperature for 1 h and then at 4 °C overnight. Blood samples were centrifuged at 2000× *g* for 5 min. Sera were collected and frozen at –80 °C for further use. Individual tissue samples were fixed in RNAlater (Sigma-Aldrich, Steinheim, Germany) overnight at 4 °C and stored at −20 °C until further processing for RNA isolation.

### 2.2. Parasite Detection

Blood smears were prepared on clean microscopic slides, stained with Diff-Quick stain (Labor+ Technik, Goerzallee, Berlin, Germany) and examined by light microscopy. Additionally, the parasite presence in blood was confirmed with nested PCR as described earlier [6]. Furthermore, histology was performed following the method by Kumar et al. [6]. Briefly, tissues were fixed in 10% neutral buffered formalin followed by washing, dehydration and embedding in paraffin wax. Tissue sections of 5 µm were prepared and either stained with Haematoxylin and Eosin or further processed for immunostaining. Immunostaining was carried out using *Tetracapsuloides bryosalmonae* monoclonal antibody P01 (Aquatic Diagnostics, Stirling, UK) following the manufacturer’s recommendations. A VECTASTAIN^®^ ABC HRP Kit (Vector laboratories, Burlingame, CA, USA) was used to visualise the antibody–antigen reaction. Afterwards, sections were counterstained with haematoxylin, mounted, and examined.

### 2.3. Parasite Antigen Preparation

To optimise the indirect enzyme-linked immunosorbent assay (ELISA), parasite antigen was prepared. For this purpose, a large number of parasite sacs (*n* = 10,000) was collected from the laboratory infected bryozoan *Fredericella sultana*. The parasite sacs were pelleted by centrifugation at 4500× *g* for 5 min and resuspended in 800 µL of TE buffer. Sample was then homogenised using tissue lyser II (Qiagen) for 2 min at 25 Hz. Subsequently, the sample was freeze–thawed in liquid nitrogen (6X) followed by six rounds of sonication on ice for 10 s at 10 Hz (40% power). Later, the sample was centrifuged at 18,000× *g* for 5 min at 4 °C. The soluble lysate was collected and stored at −80 °C until use.

### 2.4. Immunoassay

The level of specific anti-*T. bryosalmonae* antibody in each infected brown trout serum (*n* = 6) was determined at different time points using indirect enzyme-linked immunosorbent assay (ELISA). The optimisation of the ELISA was accomplished with *T. bryosalmonae* antigen ranging from 1.0 to 10.0 µg/mL, serum dilutions from 1:20–1:800, rabbit anti-salmonid Ig antibody (Bio-Rad, product code: AHP761) dilutions from 1:1500–1:9000, and peroxidase-conjugated antirabbit IgG (whole molecule) antibody (Sigma-Aldrich) dilutions from 1:1500–1:12,000.

A 96-well microtiter plate (MaxiSorp^TM^, Nunc, TherrmoFisher Scientific, Roskilde, Denmark) was coated with 100 µL/well parasite antigen (2.0 µg/mL) in bicarbonate coating buffer (pH 9.6, Sigma-Aldrich) and incubated overnight at 4 °C. The wells were washed 3 times with phosphate-buffered saline (pH 7.4) containing 0.05% Tween 20 (PBS-T) and once with PBS and then blocked with 2% bovine serum albumin (BSA, Sigma-Aldrich) in PBS for 1 h at 37 °C. The plate was washed and then 100 µL/well of 1:40 fish serum diluted in 1% BSA was added. After incubating for 1 h at 37 °C, the plate was washed again and rabbit anti-salmonid Ig antibody (1:3000) was added to each well and incubated for 1 h at 37 °C. After the washing, 100 µL peroxidase-conjugated antirabbit IgG antibody (1:6000) were added to each well and incubated for 1 h at 37 °C. After the last washing, TMB peroxidase substrate (Sigma-Aldrich) was added and incubated at room temperature for 15 min. The reaction was stopped with stop reagent (Sigma-Aldrich), and the plate was read at 450 nm with plate reader. Wells with all antibodies and substrates were included as negative controls for the ELISA. The test on each serum sample was conducted in triplicate. The mean values of antibody levels (*n* = 6) and parasite intensity (as measured below in Section 2.7) in infected brown trout were log2 transformed at each time point. Further, correlations between antibody levels in serum and parasite intensity in organs were evaluated using Pearson’s product–moment correlation coefficient (R) in R (version 1.2.5033).

### 2.5. RNA Extraction

The RNeasy Mini Kit (Qiagen, Hilden, Germany) with an on-column DNase digestion step was used to extract total RNA from the posterior kidney samples of exposed (*n* = 6) and unexposed control (*n* = 6) brown trout according to manufacturer’s protocol. Initially, individual tissue samples (10 mg) were lysed in RLT buffer containing β-mercaptoethanol. Following this, steel beads were added to the sample and homogenised using TissueLyser II (Qiagen) for 2 min at 20 Hz. Finally, the quality and amount of total RNA extracted were determined by NanoDrop 2000 spectrophotometer and by 4200 TapeStation (Agilent, Santa Clara, CA, USA). Afterwards, reverse transcription was performed using iScript cDNA Synthesis Kit (Bio-Rad, Hercules, CA, USA) on one µg of total RNA isolated from infected and control posterior kidney samples.

### 2.6. Primer Designing

Based on the kidney transcriptome data (NCBI Bioproject ID PRJNA542491) [36], we selected some B-cell-associated genes that showed differential expression (Table 1) to understand the serum antibody/B-cell-associated response against *T. bryosalmonae*.

The genes analysed for their expression in this study include C4A (Complement component 4); CD34 (Haematopoietic progenitor cell antigen CD34-like); CD79A (B-cell antigen receptor complex-associated protein alpha chain-like); CD74 (H-2 class II histocompatibility antigen gamma chain-like); BLNK (B-cell linker protein-like); BCL7(B-cell CLL/lymphoma 7 protein family member B-A-like) and CD22 (B-cell receptor CD22-like). Specific primers were designed for these genes using Primer blast tool from NCBI. The optimum annealing temperature of the designed primers was determined by gradient PCR and primer efficiency of each gene was checked using serial dilutions. Further, unique amplicon of each primer set was sequenced and BLAST analysed to ensure their specificity and sensitivity.

### 2.7. Reverse Transcription-Quantitative Real Time PCR (RT-qPCR) Analysis

The real-time PCR assay for every gene was performed in a total volume of 10 µL containing 5 µL of 2× SsoAdvanced™ Universal SYBR Green Supermix (Bio-Rad), 0.5 µL of forward and reverse primer, 1 µL of nuclease free water and 3 µL of 1:20 dilution of cDNA samples. The real-time qPCR cycling conditions included initial denaturation at 95 °C for 5 min, followed by 37 cycles of final denaturation at 95 °C for 30 s, annealing at 60, 64 and 66 °C for 30 s (annealing temperature was different for primers, presented in Table 1) and elongation at 72 °C. Final elongation was performed at 95 °C for 30 s. Melting curve analysis was carried at 60–90 °C with an increment of 0.5 °C per 10 s. The real-time PCR was performed on CFX96 Touch Real-Time PCR detection system (Bio-Rad, München, Germany). The relative parasite intensity was measured using the mean Cq values of six individual samples at each time point by qPCR using *T. bryosalmonae* 60S ribosomal protein L7 (RPL7) gene. The expression of host target genes and parasite gene was normalised to the geometric mean of both reference genes: elongation factor alpha and β-actin [35]. The relative gene expression of host genes between exposed and control groups was calculated using 2 − ΔΔCt method [37].

The two-tailed unpaired Student’s t-test with Welch’s correction was employed for analysing the statistical significance of the difference between the groups. Relative host gene expression levels were Log2 transformed and its correlations with *T. bryosalmonae* intensity was analysed using Pearson product–moment correlation coefficient (R) in R (version 1.2.5033).

## 3. Results

### 3.1. Clinical Signs

**Figure 1:**
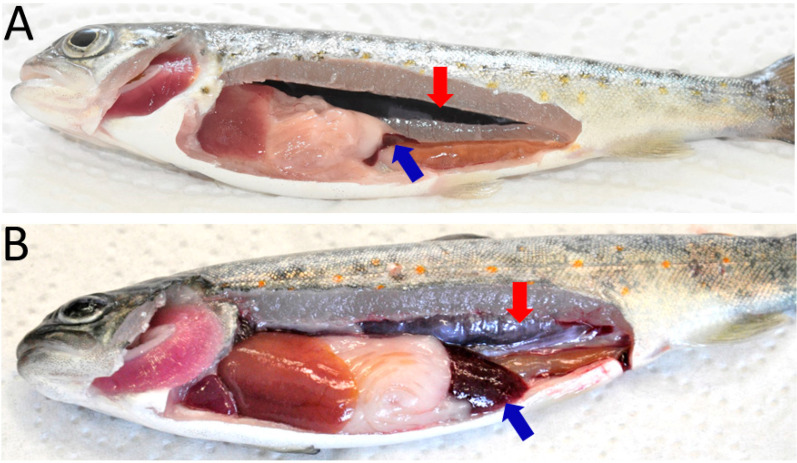
Clinical manifestations of proliferative kidney disease in brown trout. (**A**) Control fish showing normal kidney and spleen; (**B**) infected fish showing renal hyperplasia (enlargement of kidney) and splenomegaly (enlargement of spleen). Red arrow: kidney, blue arrow: spleen.

Typical clinical symptoms of PKD such as renal hyperplasia, splenomegaly and pale liver were observed at 6, 8, 10, and 12 wpe (Figure 1). However, none of the exposed fish exhibited clinical signs at 2, 4, and 17 wpe. No clinical symptoms were observed in unexposed control fish.

### 3.2. Parasite Detection

**Figure 2:**
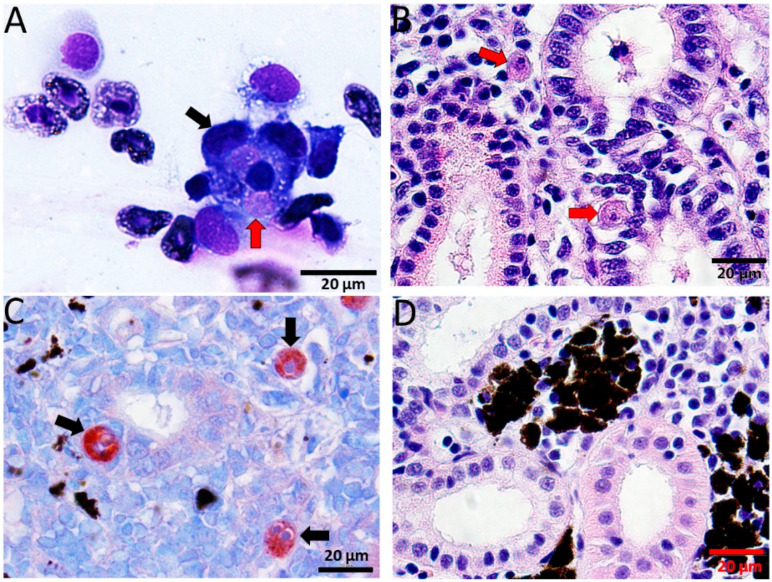
Diff-Quick stained blood smear and histological sections of kidney of infected brown trout. (**A**) Macrophages (black arrow) attached to the *T. bryosalmonae* (red arrow); (**B**) H&E stained sections showing presporogonic stages of the parasite (red arrows) in the renal interstitium and renal tubule; (**C**) immunostained sections showing parasitic stages in renal interstitium and tubular epithelium (black arrows); (**D**) H&E stained sections from control kidney showing normal renal structure.

*T. bryosalmonae* was detected in blood smears from 2 to 6 wpe (Figure 2A), which was confirmed by nested PCR (data not shown). Furthermore, histological examination showed the presence of presporogonic parasite stages in the kidney (Figure 2B,C). The degeneration of kidney tubules, necrosis and reduction of melanomacrophages in the kidney were evident from histological examination of exposed brown trout mainly from 6 to 12 wpe.

Immunohistochemical staining of kidney sections revealed *T. bryosalmonae* stages from 4–12 wpe (Figure 2D).

### 3.3. Anti-T. bryosalmonae Antibody Response

**Figure 3:**
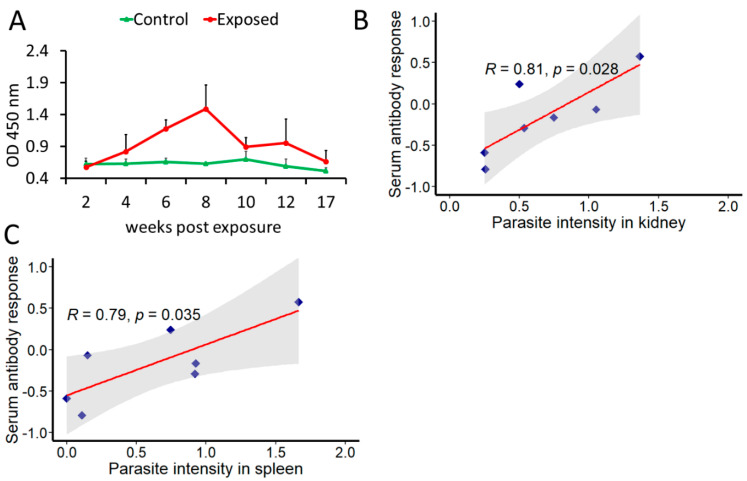
Determination of anti-*T. bryosalmonae* antibody and its correlation with parasite intensity. The mean values of antibody levels and parasite intensity in infected brown trout (*n* = 6) were log2 transformed (**A**) Kinetics of anti-*T. bryosalmonae* antibody in brown trout using indirect ELISA at different time points; (**B**) correlation between serum antibody levels and parasite intensity in kidney; (**C**) correlation between serum antibody levels and parasite intensity in spleen. For correlation plots, values on x and y axes are log2 transformed. Pearson product-moment correlation coefficient (R) was calculated and statistical significance was tested at *p* = 0.05. Shaded region represents 95% confidence intervals.

The kinetics of serum antibody response to *T. bryosalmonae* antigen is shown in Figure 3A. First anti-*T. bryosalmonae* antibody response was detected at 4 wpe (OD = 0.82). Thereafter a progressive increase was observed and the peak was observed at 8 wpe (OD = 1.49). At 10 wpe, the antibody levels declined (OD = 0.89) followed by a transient increase before decreasing at 17 wpe (OD = 0.66).

A positive correlation between parasite intensity and antibody levels was found in both kidney (R = 0.81, *p* = 0.028) and spleen (R = 0.79, *p* = 0.035) (Figure 3B,C).

### 3.4. Gene Expression in Kidney and Spleen

**Figure 4:**
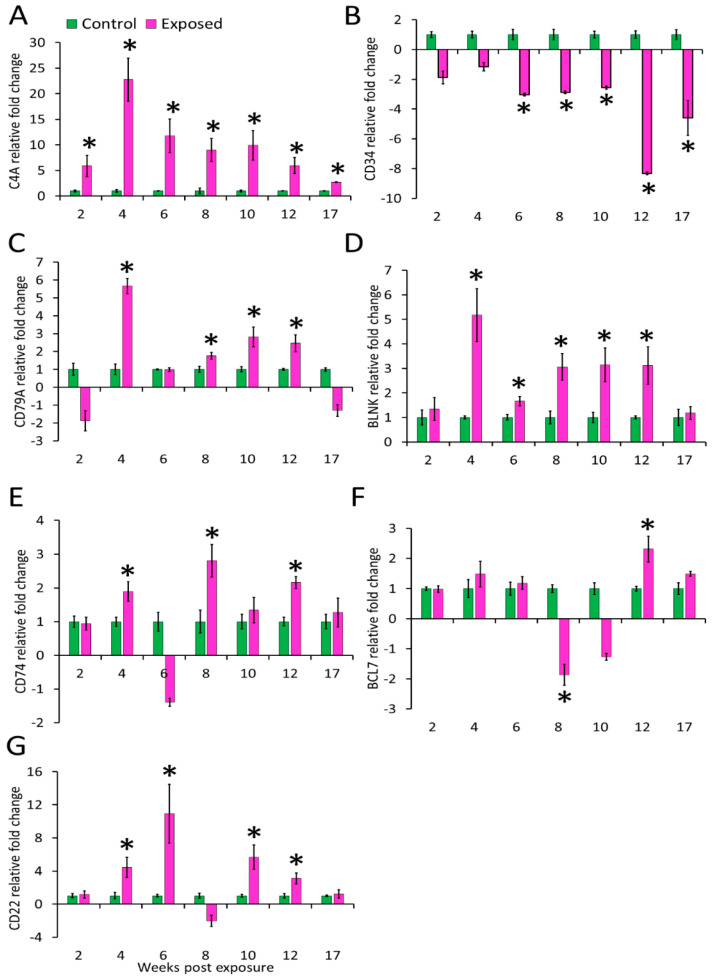
Relative expression of immune genes in kidney. (**A**) C4A; (**B**) CD34; (**C**) CD79A; (**D**) BLNK; (**E**) CD74; (**F**) BCL7; and (**G**) CD22. The relative fold change was first normalised to EF-1 and β-actin, and then represented as a fold change relative to control fish gene expression levels. Asterisks (*) denote the significant difference in relative fold change expression. Each bar shows the mean ± SEM (*n* = 6).

**Figure 5:**
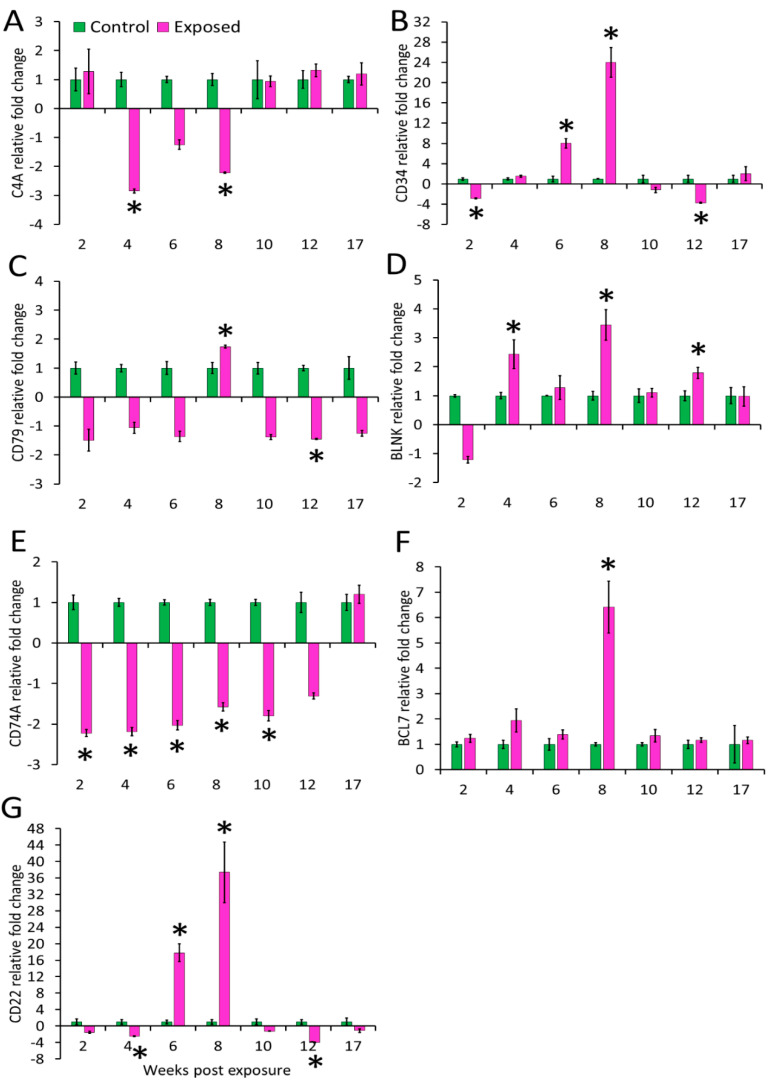
Relative expression of immune genes in spleen. (**A**) C4A; (**B**) CD34; (**C**) CD79A; (**D**) BLNK; (**E**) CD74; (**F**) BCL7, and (**G**) CD22. The relative fold change was first normalised to EF-1 and β-actin, and then represented as a fold change relative to control fish gene expression levels. Asterisks (*) denote the significant difference in relative fold change expression. Each bar shows the mean ± SEM (*n* = 6).

The expression profiles of the seven genes (complement cascade gene C4A and B-cell-associated genes) in posterior kidney and spleen tissues of infected and control brown trout are represented as relative fold change (Figure 4 and Figure 5). C4A was upregulated in the kidney at all time points from 2–17 wpe. The highest upregulation occurred at 4 wpe (22.8 folds). On the contrary, in spleen C4A was downregulated at 4 wpe (−2.8 folds) and 8 wpe (−2.21 folds) and no significant change in expression occurred at all other times.

The expression of CD34 in kidney was decreased at all time points from 6–17 wpe with the highest decrease at 12 wpe (−8.32 folds) and no significant difference in expression was observed at 2 and 4 wpe. In the spleen its expression was increased at 6 wpe (8 folds) and 8 wpe (24 folds) and decreased at 2 (−2.9 folds) and 12 wpe (−3.7 folds) while expression did not alter at rest of the time points. CD79A was upregulated at majority of the time-points (4, 8, 10, and 12 wpe) in the kidney and at other time points, change in expression was not significant. The highest expression of CD79A (6 folds) occurred at 4 wpe. In the spleen, CD79A was found to be significantly upregulated at 8 wpe (1.74 folds) and downregulated at 12 wpe (−1.5 folds). However, the expression of CD79A was not significant at 2, 4, 6, 10, and 17 wpe. CD74 showed a comparable increase at 4, 8, and 12 wpe in the kidney whereas in the spleen its expression was decreased from 2 to 10 wpe.

The B-cell signaling pathway genes were variably expressed during the course of infection. Among the studied genes, BCL7 expression decreased at 8 wpe (−1.9 folds) and increased at 12 wpe (2 folds) in the kidney. However, in the spleen this gene significantly expressed only at 8 wpe (6 folds). BLNK was upregulated from 4 to 12 wpe in kidney and at 4, 8, and 12 wpe in the spleen of exposed brown trout.

In the kidney of exposed group, CD22 was upregulated at 4, 6, 10, and 12 wpe and downregulated at 8 wpe. While in spleen, its expression was decreased at 4 and 12 wpe and increased at 6 and 8 wpe.

**Figure 6:**
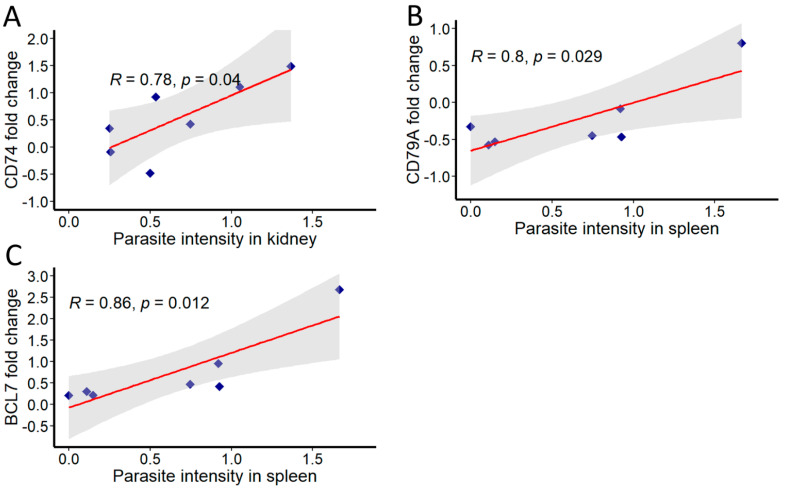
Correlation of *T. bryosalmonae* intensity and immune genes in infected brown trout kidney and spleen. The mean values of fold change and parasite intensity in infected brown trout (*n* = 6) were log2 transformed and correlation coefficient (R) was determined using Pearson product-moment at each time point. Pearson product-moment correlation was calculated and statistical significance was tested at *p* = 0.05. Shaded region represents 95% confidence intervals. (**A**) CD74, (**B**) CD79A and (**C**) BCL7.

Further, correlations were analysed between the parasite intensity and expression of immune genes in both kidney and spleen at different time points (Figure 6). In kidney, significant correlation was observed with parasite intensity only for the expression of CD74 (R = 0.78). In spleen, genes found to be correlated include CD79A (R = 0.8) and BCL7 (R = 0.86). All correlations were considered significant at *p* < 0.05.

## 4. Discussion

*T. bryosalmonae* invade fish primarily through the gills and, are later transported to the main target organ kidney through the blood [10]. Earlier studies reported the parasite in blood of rainbow trout at 4 wpe [11] and in wild brown trout [38]. In our study, parasite presence was detected in blood by smears and nested PCR from 2 to 6 wpe (preclinical phase) but not from 8 to 10 wpe (clinical phase) suggesting that the parasites migrate from blood to the organs such as kidney and spleen. Additionally, in the blood of infected brown trout, the parasite was detected by PCR at later time points such as 104 wpe, when there were no clinical signs of the disease [12]. This indicates that the parasite might be present in the blood only during the pre- and post-clinical phases of the disease. The presence of parasite during the post-clinical phase may be due to parasite replication. However, precisely the underlying reason is not yet clear. Further studies focused on in vivo imaging techniques might play a key role in delineating the route of parasite migration from the time of entry in fish and, also for exploring other aspects of interaction between host and parasite. Similar in vivo imaging technology led to the discovery of novel sites of infection with *Trypanosoma cruzi* in murine model [39].

Kidney being the major target organ of *T. bryosalmonae* presents the most prominent disease manifestation. However, *T. bryosalmonae* reportedly invade spleen and other organs as well. In line with earlier reports, we observed swelling in the kidney and spleen from 6 to 12 wpe in the exposed brown trout and hence our study focused on these two major target organs. Previous studies have reported clinical signs mostly from 6 to 10 wpe [12], and 8 to 17 wpe in brown trout [6], and in rainbow trout from 6 wpe [47 days post exposure (dpe)] to 10 wpe (75 dpe) [40], and 8 to 14 wpe [6]. The parasite proliferation triggers intense granulomatous cellular reaction, finally causing swelling in these organs [41,42]. After resolution of clinical symptoms in terms of normal organ structure restoration is confirmed in surviving brown trout [7,12,43] and rainbow trout [33]. In the present work, we did not observe clinical signs at 17 wpe, indicating that the fish had recovered and the damage to the affected organs reversed. In contrast to rainbow trout, where parasite clearance is reported, the brown trout continue to release viable spores probably throughout their lives. However, the mechanism of recovery in both hosts as well as parasite clearance in rainbow trout is not understood entirely. Bailey et al. [44] proposed a few possible explanations for recovery from clinical disease and parasite clearance in rainbow trout. One proposition by the authors was regarding the role of B cells in parasite clearance and recovery of clinical symptoms via restoration of B-cell homeostasis, with the other being the degradation of the parasite, resulting in termination of life cycle in rainbow trout.

Vertebrate hosts respond to parasitic infections by generating antibodies, which are effector molecules of humoral immunity. Antibody-mediated immune response plays a critical role during many parasitic infections to protect, clear parasite or to confer resistance to fish [45]. Antiparasite antibodies have been reported in fish during certain parasitic infections such as *Bothriocephalus acheilognathi* [46], *Trypanosoma sp*. [47] and *Neobenedenia melleni* [48]. Anti-*T. bryosalmonae* antibodies have been detected in the sera of rainbow trout at six wpe by indirect immunofluorescence testing [49], however, this test does not provide quantitative measurement of antiparasite antibody. In the present study, we found the anti-*T. bryosalmonae* antibody levels in the serum of exposed brown trout from 4 to 17 wpe. While in the early phase of infection antibodies progressively increased, they declined in the late phase. The persistence of antibodies even at 17 wpe probably indicates a continuous stimulation of immune system due to parasite presence. Additionally, antiparasitic antibodies were also found to have a positive correlation with parasite intensity in the kidney and spleen. This suggests that antiparasite antibody response during the course of *T. bryosalmonae* infection might be useful for profiling PKD-associated clinical manifestations and developing vaccines, as well as for indicating if the host has mounted an effective humoral response. In fish, three isotypes or classes of antibodies (IgM, IgD, and IgT), while in mammals, five isotypes namely IgM, IgG, IgD, IgT, and IgA are known [50]. Considerable progress has occurred in the field of fish immunology and much of the knowledge derives from analogy to higher vertebrates. In many mammals, the production of antibody isotypes depends on the mode of B-cell activation and the inflammatory environment surrounding the B-cell subsets is established [51]. For example, during *Plasmodium* infection, the proinflammatory Th1 environment primarily promotes isotype flipping to cytophilic or opsonising Abs, such as IgG1 and IgG3 in humans [52] and IgG2a and IgG2b in mice [53]. Research has elucidated significant upregulation of Th1-like cytokines during PKD in brown trout at day 50 post exposure to parasite [33]. In the present study, maximum antibody titre was observed around the same time, i.e., at 8 weeks post exposure. It is noteworthy that fish do not possess IgG though IgM is known for opsonising pathogens [54]. Similar to mammals, fish antibody isotypes are known to play specific roles, however, not much has been elucidated. In this context, the confirmation of the type of immunoglobulin present at this time point could help in gaining insights into the generated immune response.

While our study chronicles the systematic antiparasite antibody response in *T. bryosalmonae*-exposed brown trout, it has a potential caveat. As previously stated, we used antigens prepared from parasite sacs developed in bryozoans, which are infective for fish. The stages of *T. bryosalmonae* have distinct attributes in primary (bryozoan) and secondary (salmonid) hosts. For instance, *T. bryosalmonae* spores have one amoeboid cell and two polar capsules in bryozoans and two amoeboid cells and four polar capsules in fish [55]. Inside the fish, *T. bryosalmonae* undergo developmental transition through presporogonic and sporogonic stages. Consequently, the response of antibodies targeting presporogonic and sporogonic parasite stages derived from fish might differ, relative to the infective stages derived from bryozoans, due to their different antigenic determinants. Thus, it is plausible that the antibody levels observed may differ little from the parasite antigens derived from kidney in infected brown trout. Saulnier and de Kinkelin [56] demonstrated the antigenicity of parasite antigens prepared from infected kidney towards sera of *T. bryosalmonae* infected rainbow trout during the development of monoclonal antibodies against *T. bryosalmonae* but they did not examine the systematic antiparasite antibody response in infected fish. Testing the sera against homogenates of severely infected fish tissues could have been one approach to address this possibility. Additionally, further study is needed to investigate the extent of antigenic differences present between parasite derived from bryozoans and parasite derived from kidney.

Although antibodies by themselves can act on parasites through multiple mechanisms, complement activation enhances their activity. Considering this, we investigated the expression of the complement cascade gene C4A. As expected, the upregulation of this gene was evident at all the studied time points in the kidney. The constant upregulation of C4A probably reflects the importance of a complementary pathway in *T. bryosalmonae* infection. Together with C3A and C5A, C4A leads to complement pathway activation [57]. Complement is amongst the first line of defence in fish hosts and is involved in inflammatory response and pathogen killing through opsonisation. Activation of the complement cascade stimulates degranulation of basophils and mast cells and increases vascular permeability. In the present study, expression pattern of C4A points toward its possible role in persistent vascular permeability for the continued infiltration of immune cells into the kidney during chronic infection. On the contrary, in the spleen significant downregulation occurred at 4 and 8 wpe, whereas at all other time points no significant difference was observed between exposed and control fish.

We also investigated the expression of CD34, which is a marker of haematopoietic stem cells (HSCs). This gene was predominantly downregulated (6 to 17 wpe) in the kidney whereas in the spleen downregulation was noticed during early (2 wpe) and late stage (12 wpe). CD34 was upregulated at 6 and 8 wpe with highest expression (24 folds) at 8 wpe in the spleen. CD34 plays an important role in differentiation of haematopoietic stem and progenitor cells. The downregulation of this gene promotes the differentiation of HSCs into granulocytes and megakaryocyte lineages and suppresses erythroid lineages [58]. This might be the underlying reason for the presence of inflammatory cells in the posterior kidney of exposed brown trout as also reported in previous studies.

Expression of genes associated with B cell was analysed to understand B-cell-mediated immune response during PKD. B lymphocytes are key players in the immune response against pathogens, particularly through their role in antigen presentation, secretion of cytokines and antibody production in all vertebrates, including fish. Upon antigen contact, BCR are activated and initiate a cascade of processes leading to antibody production. B cells are characterised by the presence of surface markers CD34, CD79 and CD22. Recently, CD79 and CD34 were identified as B-cell markers in salmon [59]. CD79A is a component of the CD79 transmembrane protein. Along with surface immunoglobulins, CD79 forms the signalling unit of B-cell antigen receptor complex for B-cell growth, differentiation and proliferation [60]. In our study, CD79A was upregulated in the kidney at most time points, whereas in spleen upregulation was observed at 8 wpe and downregulation at 12 wpe. Similar CD79A upregulation in fish kidney at early stages and in spleen at later stage is reported during *C. irritans* infection, possibly an indication of B-cell differentiation in the kidney and maturation in the spleen [61].

Many cells including B and T-cells, macrophages, epithelial and endothelial cells express CD74 (MHC class II invariant chain), which is a type II transmembrane glycoprotein [62]. It has been reported from many teleost fish and supposedly functions similar to that in mammals [63]. In this study, CD74 was upregulated in the kidney whereas it was downregulated in the spleen of exposed brown trout. Among the multitude of functions, CD74 serves an important role in inflammation and tissue repair in mammals. CD74 is the receptor of the inflammatory cytokine MIF (macrophage migration inhibitory factor) which regulates the movement of activated immune cells to the site of inflammation [64]. Furthermore in mammals, another member of MIF family, MIF-2, is described, which binds to CD74 and initiates signalling pathways promoting tubular cell regeneration and helps in recovery of injured kidney tissue [65]. Besides, MIF is also known to engage other chemokine receptors including CXCR2 [66], which was significantly upregulated from grade 1 to grade 3 swollen kidneys in PKD-infected rainbow trout [67]. The upregulation of CD74 during the clinical phase of PKD in brown trout, marked by intense inflammatory response, followed by recovery of the kidney tissue might be indicative of a similar role in this fish host. Additionally, CD74 expression in cells increases in response to IFN-γ [68]. The upregulation of interferon-γ in the posterior kidney of brown trout during *T. bryosalmonae* proliferation is reported [33,34,35,36]. Nevertheless, more studies are warranted to confirm the role of CD74 during PKD in exposed fish.

B-cell CLL/lymphoma 7 protein family member B-A-like gene (bcl7ba) is a member of the BCL7 gene family. Although this gene has been characterised from many fish species including trout, which is evident from the sequences available in the NCBI database, functional studies are lacking. In *Caenorhabditis elegans,* BCL-7 regulates terminal cell differentiation in somatic stem-like cells, whereas in human gastric cancer cells it functions to positively regulate apoptosis by inhibiting the expression of antiapoptotic factors [69]. Interestingly, in our study, this gene was significantly downregulated at 8 wpe (−1.9 folds) and upregulated at 12 wpe (2.3 folds) in the kidney. A probable explanation for this effect, could be that this gene has a role in tissue regeneration during recovery following *T. bryosalmonae* infection [41]. In the spleen, it was found to be highly upregulated (6.4 folds) only at 8 wpe in the spleen. The genes of BCL7-family members share a unique N-terminal domain that has been evolutionarily conserved in the animal kingdom, but the remaining sequences lack homology [70]. The results from this study are indicative of an important role of BCL7 family genes during PKD pathogenesis and hence should be studied in greater depth.

The adaptor protein, BLNK, functions in the BCR signalling pathway [71]. This protein is involved in the activation of B-cell receptor-associated kinase to downstream signalling pathways, affecting B-cell activation [60]. We observed significant upregulation of this gene in the kidney from 4 to 12 wpe and in spleen at 4, 8, and 12 wpe. BLNK regulation in different organs was reported following *C. irritans* infection. Mo et al. [72] reported the differential regulation of BLNK in head kidney, spleen, skin and gill tissues. They suggested proliferation and differentiation of B cells and macrophages in the primary haematopoietic organ, followed by migration to infected sites.

Significant upregulation of CD22 was evident at 4, 6, 10, and 12 wpe in infected kidney. In the spleen at early (4 wpe) and late time points (12 wpe) it was significantly downregulated whereas at 6 and 8 wpe it showed upregulation. CD22 is a lectin-like member of the Ig superfamily expressed exclusively by mature B cells. CD22 acts as an antagonist to B cell activation most likely by enhancing the threshold of BCR-induced signals [73]. Upregulation of CD22 has been demonstrated in skin during *C. irritans* infection in *Epinephelus coides* [74].

Given the important role of B cells in modulating the fate of *T. bryosalmonae* and its pathogenesis in the host, we examined the correlation between the parasite intensity and the expression of the above-discussed B-cell-associated genes in kidney and spleen. Overall, parasite intensity in kidney significantly correlated only with CD74 expression. Likewise, the expressions of CD79A and BCL7 showed a positive association with parasite intensity in spleen. As discussed previously, the positive correlation of CD74 probably reflects its important role in controlling parasite burden by mediating inflammatory response in the kidney. Similarly, the positive associations of CD79A and BCL7 indicate that these genes play a crucial role in the defence against the parasite in spleen.

## 5. Conclusions

Our study demonstrates the kinetics of anti-*T. bryosalmonae* antibody response against parasite sac antigens in chronically infected brown trout for the first time. The observed trend of parasite-specific antibodies in the sera and presence of parasites in the kidney of infected fish point towards a sustained activation of the immune system. Though this study generates significant information, there could be a possible extension to our work, wherein antibodies specific to various developmental stages of *T. bryosalmonae* in fish can be studied to gain enhanced understanding of the specific humoral response against this parasite of economic and ecological relevance. The kinetics of a parasite-specific antibody response may serve as markers of protection and thus may be important for vaccine development. We also investigated the expression pattern of complement cascade gene C4A and some B-cell-associated genes for the first time during PKD pathogenesis, which provides valuable insights into the observed inflammation, host survival and recovery. The antibody and B-cell-mediated response observed in this study reflect a strategy of parasite persistence and host survival. Although the differential modulation of these genes in kidney and spleen in course of the infection suggest an important role of these genes in immunopathogenesis, more studies are required to define the role of B cells which might be helpful in developing therapeutic strategies for controlling *T. bryosalmonae* infections.

## Figures and Tables

**Table 1:** List of quantitative qRT-PCR primers used in this study. F: forward; R: reverse; bp: base pairs.

Code	Sequence	Size (bp)	Annealing (°C)	Primer Efficiency
C4A F	CTGCCCACTCTGTGTCCTTA	161	64	93.1
C4A R	GGCAACTGAAGGGAAAGACC			
CD34 F	GTGTGTGCGTCAGCTATACA	195	60	93.0
CD34 R	GATCTGGGTTCAGCTTGCAG			
CD79A F	GAGTGGACCGGAGAGACAAC	185	66	96.0
CD79A R	GTAGACATGCAGGAAGGTGC			
CD74 F	ACGAAAAGACTCCCATGACG	144	60	95.0
CD74 R	TCCATCTGTCTCTTCAGGCT			
CD22 F	GTCCAACTCTCCTAACCGCT	191	60	90.0
CD22 R	CAGCAGGTAGGGCTCTAGTC			
BCL7 F	GAAGGTCATGGCGGTCATTG	196	60	95.0
BCL7 R	GTGTGGGTTTTCTGAGGCTG			
BLNK F	TATCATTGGCACTTTGCCCAG	188	60	93.0
BLNK R	GGCTGAACATGCCTTACACC			
RPL7 F	GATTAGGATATCCCCAAGCAACG	152	60	92.5
RPL7 R	AGGTATTCCTCATGTACCTCCAA			
EF alpha F	AGACAGCAAAAACGACCCCC	167	60	90.3
EF alpha R	AACGACGGTCGATCTTCTCC			
β-actin F	CAGGCATCAGGGAGTGATG	127	60	96.5
β-actin R	GTCCCAGTTGGTGACGATG			
